# Differential Activity of Voltage- and Ca^2+^-Dependent Potassium Channels in Leukemic T Cell Lines: Jurkat Cells Represent an Exceptional Case

**DOI:** 10.3389/fphys.2018.00499

**Published:** 2018-05-09

**Authors:** Salvador Valle-Reyes, Georgina Valencia-Cruz, Liliana Liñan-Rico, Igor Pottosin, Oxana Dobrovinskaya

**Affiliations:** Centro Universitario de Investigaciones Biomédicas, Universidad de Colima, Colima, Mexico

**Keywords:** calcium signaling, potassium channel, voltage gating, current density, T leukemia, T lymphocyte

## Abstract

Activation of resting T cells relies on sustained Ca^2+^ influx across the plasma membrane, which in turn depends on the functional expression of potassium channels, whose activity repolarizes the membrane potential. Depending on the T-cells subset, upon activation the expression of Ca^2+^- or voltage-activated K^+^ channels, KCa or Kv, is up-regulated. In this study, by means of patch-clamp technique in the whole cell mode, we have studied in detail the characteristics of Kv and KCa currents in resting and activated human T cells, the only well explored human T-leukemic cell line Jurkat, and two additional human leukemic T cell lines, CEM and MOLT-3. Voltage dependence of activation and inactivation of Kv1.3 current were shifted up to by 15 mV to more negative potentials upon a prolonged incubation in the whole cell mode and displayed little difference at a stable state in all cell lines but CEM, where the activation curve was biphasic, with a high and low potential components. In Jurkat, KCa currents were dominated by apamine-sensitive KCa2.2 channels, whereas only KCa3.1 current was detected in healthy T and leukemic CEM and MOLT-3 cells. Despite a high proliferation potential of Jurkat cells, Kv and KCa currents were unexpectedly small, more than 10-fold lesser as compared to activated healthy human T cells, CEM and MOLT-3, which displayed characteristic Kv1.3^high^:KCa3.1^high^ phenotype. Our results suggest that Jurkat cells represent perhaps a singular case and call for more extensive studies on primary leukemic T cell lines as well as a verification of the therapeutic potential of specific KCa3.1 blockers to combat acute lymphoblastic T leukemias.

## Introduction

Ion channels are pore-forming proteins that mediate transport of ions across biological membranes. A cell of any type is characterized by a specific pattern of ion channels differentially expressed at various physiologic conditions and organized in precisely regulated functional network. Potassium (K^+^)-selective channels are key elements involved in control of membrane potential, cell volume regulation, and shaping of Ca^2+^ signal (extensively reviewed by Cahalan and Chandy, 2009; Feske et al., 2012).

In T cell biology, the most-studied event is activation of naïve or memory T cells through TCR/CD3 complex, leading to proliferation, clone expansion and differentiation into effector T cells. It was estimated that 75% of all genes upregulated during antigen activation in T cells are dependent on Ca^2+^ influx (Feske et al., 2001).

Principal Ca^2+^ influx in T cells involves a coordinated interplay of several channels (reviewed by Feske, 2013). Initially, hydrolysis of inositol triphosphate and activation of inositol triphosphate receptors provokes Ca^2+^ release from intracellular stores, mainly from the endoplasmic reticulum. Depletion of Ca^2+^ in the endoplasmic reticulum causes conformational changes in Ca^2+^ sensor protein STIM, its interaction with plasma membrane protein ORAI, and formation of the functional complex ORAI/STIM, habitually known as Ca^2+^ release- activated Ca^2+^ channel (CRAC). CRAC appears to be a core element for Ca^2+^ influx during T cell activation. Due to an intrinsic inward rectification, CRAC-mediated Ca^2+^ influx is strongly potentiated by membrane hyperpolarization, while depolarization reduces Ca^2+^ entry. Whereas Ca^2+^ influx *per se* causes the plasma membrane depolarization, K^+^ efflux is required to maintain a hyperpolarized membrane potential critical for sustained, long-lasting Ca^2+^ increase indispensable for new gene expression (reviewed by Feske et al., 2012). In healthy T cells two distinctly regulated K^+^ channels control the plasma membrane potential, voltage-activated Kv1.3 and Ca^2+^-activated intermediate-conductance KCa3.1 ones (Leonard et al., 1992; Mello de Queiroz et al., 2008).

Kv1.3 is steeply activated by a depolarization above -60 mV. At resting membrane potential around -50 mV only a tiny fraction of Kv1.3 channels is open. To achieve lower membrane potentials down to K^+^ equilibrium (∼-80 mV), the activity of some voltage-independent K^+^ channels needs to be involved. In T lymphocytes, the activation of KCa3.1 channels provokes a stable hyperpolarization, indispensable for a durable Ca^2+^ entry. Remarkably, all three channels, CRAC, Kv1.3 and KCa3.1, were shown to be recruited to and stabilize the immunological synapse during the antigene presentation (Panyi et al., 2004; Nicolaou et al., 2007; Lioudyno et al., 2008). Their co-localization is certainly required for functional interaction. Quiescent mature human T cells express predominantly Kv1.3, several hundred copies per cell, and just few copies of KCa3.1. Following activation, to sustain the more intense Ca^2+^ influx, a transcriptional upregulation of K^+^ channels occurs, and, what is remarkable, of KCa3.1 to a much larger degree than of Kv1.3. In human helper (CD4^+^) T cells, pharmacological inhibition of both Kv1.3 and KCa3.1 channels was reported to suppress the Ca^2+^ rise, causing antiproliferative effect and subsequent decrease in interleukin Il-2 production. Basing on their crucial importance in T cell activation, both Kv1.3 and KCa3.1 were proposed as drug targets for immunomodulatory therapy (Chandy et al., 2004). And then the question arises, can these channels also serve as a drug target in the case of T cell lymphoproliferative disorders, e.g., acute lymphoblastic T cell leukemias (T-ALL)? At the moment, this question could not be answered, because in contrast to healthy T cells, the data about K^+^ channels pattern and their function in leukemic T cells is mainly limited to a single T-ALL cell line, Jurkat. The reason apparently is the lack of appropriate biological material. Despite cancers demonstrate unlimited growth potential in patients, primary leukemic cells isolated directly from individuals do not survive for a long time in laboratory conditions. The factors affecting the primary cell survival include our limited knowledge about optimal culture conditions for primary leukemic cells and poor coordination between hospital and research laboratory personnel. It is worthwhile to mention here that primary leukemic cells demonstrated improved *ex vivo* survival being co-cultured with mesenchymal bone marrow stromal cells, and appropriate protocols were proposed and successfully used to maintain leukemic cells derived from patients diagnosed with B-ALL (Manabe et al., 1992), T-ALL (Winter et al., 2002), and chronic lymphocytic B leukemia (Leanza et al., 2013; Szabo et al., 2015). Nevertheless clinical samples available after the diagnostics assays, especially in the case of pediatric T-ALL patients, generally contain cells in small numbers, insufficient for systematic studies. For the same reasons, it is very difficult to establish new leukemia cell lines, where the majority of attempts failed (Drexler and MacLeod, 2003, 2010). Notably, the majority of established cell lines were derived from leukemic patients, which have previously received complex chemotherapeutic treatment in relapse, and represent the most chemoresistant aggressive clones. Consequently, the detailed characterization of the established leukemic cell lines is an important issue in search for new drugs, targeting chemoresistant clones. Apparent advantage of this approach includes the world-wide unlimited supply of identical cells providing cumulative research. Recently, a potential for such approach was demonstrated on different cancer cell lines, including acute myeloid leukemic ones, where the aberrant expression of different K^+^ channels was correlated with a drug sensitivity (Leanza et al., 2014).

Among available leukemic T cell lines, Jurkat is indisputably the best characterized. Noteworthy, Jurkat occupies particular place in T cell biology, especially in the study of TCR signaling. Indeed, many of the findings in this field were made with Jurkat cell line as the T cell *in vitro* model and then extrapolated to healthy T cells (Abraham and Weiss, 2004). With time, many researchers questioned the physiological relevance of such approach, because a number of differences in comparison to healthy cells were demonstrated for Jurkat cells (reviewed by Dobrovinskaya et al., 2015).

There are no doubts, that Ca^2+^ signaling is involved into T cell leukemogenesis. Sustained activation of Ca^2+^-dependent phosphatase calcineurin was reported both in animal models and biopsies from human lymphomas (Medyouf et al., 2007). Calcineurin activation is indispensable requirement for leukemia-initiating cell activity in T-ALL (Gachet et al., 2013; Passaro et al., 2015a,b). However, data related to functional expression of K^+^ channels involved in shaping of Ca^2+^ signal are available only for Jurkat (Fanger et al., 2001). Density of Kv1.3 channel in Jurkat is significantly lower than in human peripheral blood T cells, the fact, which is suggested by studies from multiple groups (**Supplementary Figure S1**), but up to the moment received little attention. Moreover, in Jurkat KCa3.1 channels are under-expressed and essentially replaced by small conductance KCa2.2 channels (Grissmer et al., 1992; Fanger et al., 2001). In lymphocytes, potassium channel Kv1.3 were shown to be located in sphingolipid and cholesterol-enriched membrane rafts, where lipidic environment represents important factor in regulation of ion channel activity (Bock et al., 2003). Later it was shown that experimental disintegration of lipid rafts in healthy T lymphocytes and Jurkat cells differentially modulated voltage gating of Kv1.3 channels, indicating differences in a composition of their plasma membrane microenvironment (Hajdú et al., 2003; Pottosin et al., 2007). Up to now, it remains unclear, whether these features are characteristic for T leukemia in general, or they are specific for the Jurkat cell line. The present study was designed to address this question and to compare biophysical and pharmacological characteristics as well as functional expression of Kv1.3 and KCa channels in three widely used human leukemic T cell lines, Jurkat, CEM and MOLT-3, in comparison with quiescent and activated CD4^+^ T cells, isolated from peripheral blood of non-oncologic individuals.

## Materials and Methods

### Cell Lines and Culture Conditions

All cell lines used in this study were derived from the peripheral blood of patients diagnosed with T-ALL: Jurkat cell line was established from 14-year-old boy in first relapse in 1976; MOLT-3 cell line from 19-year-old man in relapse in 1971; CCRF-CEM cell line from 3-year-old Caucasian girl in relapse (terminal) in 1964. All cell lines were purchased from ATCC, and cryopreserved in liquid nitrogen. To prevent passage-related variations in data, the cells with passage number less than 20 were used in experiments. Cells were cultured in a Roswell Park Memorial Institute 1640 (RPMI 1640) Advanced medium supplemented with 5% heat inactivated fetal bovine serum, 100 U/ml penicillin, 100 mg/ml streptomycin, 10 mM N-2-hydroxyethylpiperazine-N′-2 ethanesulfonic acid (HEPES), 2 mM glutamine (GlutaMAX^TM^ Supplement) (all from Invitrogen) at 37°C in humidified atmosphere (5% de CO_2_ and 95% air). Cells were maintained in the logarithmic growth phase by daily medium refreshment.

### Purification and Activation of CD4^+^ Lymphocytes

Mature CD4^+^ lymphocytes were obtained from peripheral blood of healthy 22–32 years old volunteers. Peripheral blood 10 ml- probes were taken by trained personal under aseptic condition with sterile material for each donor. The protocol was approved by Bioethics and Biosecurity Committee of the Biomedical Research Centre and Faculty of Medicine, in accord with Federal (Artículo 100, Ley General de Salud), State, and local laws. Informed consent was obtained in written form from all participants. Blood samples were diluted 1:1 with PBS. The mononuclear cells (CMN) were separated by Ficoll gradient (Ficoll-Paque PLUS, GE Healthcare). CMN were subjected to negative selection (to avoid activation) with CD4^+^ T cell isolation kit, following Miltenyi Biotec specifications. The CD4^+^ T cells were cultured in complete RPMI 1640 medium, in 24-wells plates (no more than 1.5 × 10^6^ cells per ml), for 24 h. For polyclonal activation, resting lymphocytes were pretreated in 96-well plates with antiCD3 monoclonal antibodies (5 μg/ml) (BD, 555336) for 2 h at 37°C; excess medium was removed and 1 × 10^5^ cells were incubated with (2 μg/ml) anti CD28 monoclonal antibodies (BD, 555725) for 4 days. Ligation of CD3/CD28 provides a powerful antigen-independent stimulus by cross-linking T cell receptor (TCR) resulting in transit from quiescent (resting) to proliferation state. This event, termed as blast formation is accompanied by a huge increase in biomass and size (Teague et al., 1993; Grumont et al., 2004; Li and Kurlander, 2010).

### Electrophysiology

Patch-clamp recordings were performed in a whole-cell configuration, immediately after gaining the access to whole cell and after at least 15 min of incubation, to evaluate initial potassium current density and voltage dependence and to measure the voltage dependence at a stable state, respectively. Patch pipettes were pulled from Kwik-Fil 1B150F-4 capillaries (World Precision Instruments, Sarasota, FL, United States) in six steps on a Brown/Flamming model P-97 puller (Sutter Instruments, Novato, CA, United States) and fire polished, using LPZ 101 microforge (List Medical, Germany). Bath solution for recording of voltage dependent potassium (Kv) channels contained (in mM): 150 NaCl, 5 KCl, 1 MgCl_2_, 2.5 CaCl_2_, and 10 HEPES NaOH (pH 7.4). For records of Ca^2+^-activated potassium (KCa) channels bath contained (in mM): 160 KCl, 1 MgCl_2_, 2.5 CaCl_2_, and 10 HEPES-KOH (pH 7.4) or, for K^+^- free condition all KCl were replaced for with equimolar NaCl. Patch electrodes for Kv records were filled with a solution containing (in mM): 134 KCl, 2 MgCl_2_, 1 CaCl_2_, 10 EGTA, and 10 HEPES–KOH (pH 7.4). For KCa records the pipette solution contained (in mM): 130 K-Aspartate, 4.75 CaCl_2_, 2 MgCl_2_, 5 EGTA, and 10 HEPES-KOH (pH 7.4, pCa 6). Liquid junction potentials (LJP) between pipette and bath solutions were calculated using the application within Clampex Version 10.3.2.1. program (Molecular Devices, LLC). Assuming that in the whole cell mode this LJP will be zeroed, instead of cancelation of the electrical potential difference between open patch-clamp electrode and the bath at the beginning of each experiment, this voltage difference was set to a respective calculated LJP value. All chemicals were of analytical quality and purchased from Sigma-Aldrich. The resistance of patch electrodes was 3–5 MΩ. Experiments were performed at 28–30°C. Current measurements were performed using an Axopatch 200A Integrating Patch-clamp amplifier (Axon Instruments, Foster City, CA, United States). Records were low-pass filtered at 2 kHz, digitized using a DigiData 1200 Interface (Axon Instruments), transferred to a personal computer, and analyzed using the pClamp 6.0 software package (Axon Instruments).

For pharmacological analysis, the following drugs were introduced by bath perfusion to a final concentration as indicated: specific Kv 1.3 blocker MgTx (1 nM), specific KCa2.2 channel blocker apamin (1 nM), specific KCa3.1 blocker clotrimazole (200 nM), and efficient blocker of Kv1.3 and KCa 3.1 channels ChTx (50 nM). All drugs were from Tocris Bioscience.

### Statistical Analysis and Data Fitting

Data are presented as means ± SE, with a number of individually tested cells, *n*, as indicated for each condition. For the comparison of the density of Kv and KCa currents statistical significance was calculated using Prism software version 6.0 (GraphPad, La Jolla, CA, United States) from adequately powered sample sizes for one-tailed tests using one-way ANOVA, unpaired Tuckey *t*-test with Bonferroni correction for multiple comparisons. Minimal criterion for statistical significance was defined as *P* ≤ 0.05. Actual *P*-values are provided in the figure legends.

Activation curves for the Kv1.3 current were fitted to a single Boltzmann function (Eq. 1):

$$\begin{array}{c} \frac{I_{tail}}{I_{tail\;\left(max\right)}}=\frac{1}{1+e^{-\frac{Z\times F\times \left(V-V_{1/2}\right)}{R\times T}}} \end{array}$$

where *Z* is a number of elementary charges to be translocated across the whole voltage drop upon the channel activation (gating charge), *V*_1/2_ is a potential, at which half of the channels turned open (midpoint potential), *V* is membrane voltage, *F* is Faraday constant, *R* is universal gas constant, and *T* is absolute temperature. In case of CEM cells, the data points were better described by a sum of two different Boltzmann functions (Eq. 2):

$$\begin{array}{c} \frac{I_{tail}}{I_{tail\;\left(max\right)}}=\frac{\theta }{1+e^{-\frac{Z_{1}\times F\times \left(V-V_{1/2}^{\left(1\right)}\right)}{R\times T}}}+\frac{1-\theta }{1+e^{-\frac{Z_{2}\times F\times \left(V-V_{1/2}^{\left(2\right)}\right)}{R\times T}}} \end{array}$$

where 𝜃 and (1-𝜃) are the weights of respective fractions. Inactivation curves can be well described by a single Boltzmann function with a small, but significant offset (*off*) (Eq. 3):

$$\begin{array}{c} \frac{I_{+50}}{I_{+50\;\left(max\right)}}=off+\frac{1-off}{1+e^{-\frac{Z\times F\times \left(V-V_{1/2}\right)}{R\times T}}} \end{array}$$

When two-pulse protocol was used to evaluate the steady-state inactivation (**Figure 2**), *I_+50_/I_+50_(max)* at the left side of the equation was substituted for *I_2nd_/I_1st_*, the amplitude ratio of Kv1.3 currents, evoked by second and first voltage steps to +20 mV.

## Results

### Kv1.3 Is a Sole Determinant of the Voltage-Dependent K^+^ Current in Leukemic T Cell Lines and Primary CD4^+^ Cells

Kv1.3 is the only member of the family of voltage-dependent Kv channels known to be expressed in healthy human T cells and Jurkat cell line (Cahalan and Chandy, 2009). Due to its characteristic pattern of time-dependent activation upon depolarization, use-dependent inactivation, and high affinity specific block by MgTx, Kv1.3 current can be with ease functionally identified in electrophysiological records. Here, we verified the identity of the Kv current in CEM and MOLT-3 leukemic T cell lines and performed an accurate comparison of its density and parameters of voltage activation and inactivation with Kv1.3 current in resting and activated helper T CD4^+^ and Jurkat cells.

**FIGURE 1:**
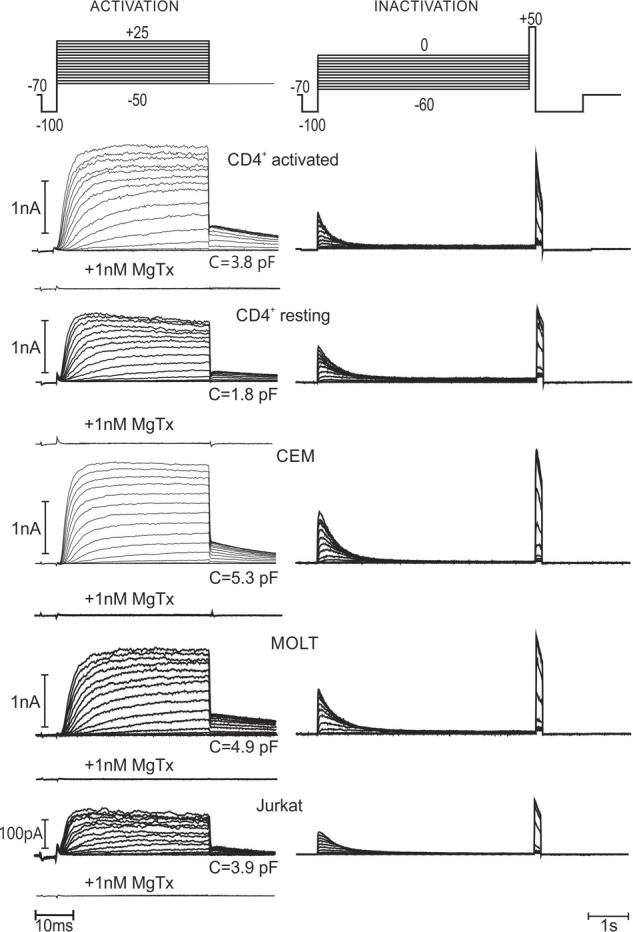
Kinetics of voltage-dependent potassium (Kv) current in human T lymphocytes and leukemic T cell lines. Typical (immediately after breaking into whole cell) current recordings, with a capacity of cell in pF given for each experiment. To activate Kv current, from the holding potential of –70 mV voltage was stepped to the values between –50 mV and +25 mV in 5 mV increments (voltage protocol and current traces at the left hand side). To evaluate the relative activation as a function of applied voltage, at the end of 37.5 ms depolarization step the voltage was switched to –50 mV, causing current deactivation; the magnitude of the tail current at the beginning of deactivation process was measured and normalized to a maximal value. To evaluate the degree of Kv inactivation as a function of applied voltage, 5 s- depolarization steps from –60 to 0 mV were applied (voltage protocol and current traces at the right hand side). The fraction of non-inactivated Kv current was estimated by a subsequent application of the voltage pulse to +50 mV and measuring the initial current amplitude. To avoid an interference with a Ca^2+^-dependent current, the pipette solution was made virtually Ca^2+^-free (8 nM Ca^2+^) by the addition of EGTA. At the end of each experiment, 1 nM margatoxin, a specific blocker of Kv 1.3 was applied to the bath, resulting in a complete block of the Kv current.

To minimize the contribution of KCa current, free cytosolic (patch pipette) Ca^2+^ was set to virtually zero (few nM). To evaluate the Kv voltage- and time-dependent activation, from a holding potential of -70 mV, shortly passing to -100 mV to verify the quality of the seal, a sequence of depolarized pulses from -50 to +25 mV was applied. The duration of test pulses was long enough to reach the maximal activation, but as short as possible to avoid a substantial inactivation. At the end of depolarizing pulse the voltage was switched to -50 mV, and, subsequently returned to a resting potential of -70 mV (**Figure 1**, left), where it was kept for 15 s to preclude a significant accumulation of inactivation from pulse to pulse. Relative magnitude of tail current at -50 mV was taken as a measure of the channel activation at the end of the test pulse (**Figure 3**, left). This voltage protocol corresponded to the beginning of experiment, immediately after gaining into whole cell configuration. As also observed by other authors (Cahalan et al., 1985; Pahapill and Schlichter, 1990), Kv1.3 voltage dependence was shifted to more negative potentials upon few minutes of incubation in the whole cell mode. Thus, the measurement was repeated after at least 15 min of incubation, but holding potential was lowered to -80 mV, depolarizing pulses were started from -60 mV and tail currents were evaluated at -60 mV for this case. Alternatively to tail current evaluation, we have also performed a more traditional analysis, measuring the voltage dependence of the peak current conductance, *G*/*G*_max_. Both approaches generally give consistent results; yet, a small but significant difference between the two datasets may be detected in case of CEM and Jurkat cells (**Supplementary Figure S2**). It should be noted that the rectification of the whole cell current may be caused not only by a voltage-dependent increase of channels’ open probability, but also by any voltage-dependent change of the single channel conductance. Therefore, the accuracy of the analysis based on the macroscopic conductance measurements depends on the correctness of the assumption of the constant single channel conductance (Ohmic behavior). Intrinsic inward rectification of the unitary Kv1.3 current appears to be “linearized” by Goldmann-type rectification due to an outward directed K^+^ gradient upon physiological conditions, resulting in a quasi-Ohmic behavior (Cahalan et al., 1985; Pahapill and Schlichter, 1990). However, some super-linear trends could be detected in unitary I/V curves, reported by Pahapill and Schlichter (1992). Thus, we preferred the tail current analysis, because the relative activity as a function of voltage may be directly read from the original recordings and the result is not dependent on the assumption of Ohmic behavior of the unitary current. At the end of each experiment, MgTx (1 nM) was added to the bath and the activation voltage protocol was run again. For all cell lines, as well as resting and activated healthy CD4+ lymphocytes, time- and voltage-dependent Kv currents were completely abolished by MgTx (**Figure 1**), which implies that the Kv current was mediated exclusively by Kv1.3 channels. It should be noted here, that MgTx at 1 nM is also a potent blocker of the Kv1.2 channels (Bartok et al., 2014). However, Kv1.2 current has biophysical properties clearly different the Kv1.3 one. In particular, it inactivates very slowly, with a characteristic time about 15 s (Sprunger et al., 1996), which is inconsistent with a relatively fast inactivation of the Kv current, reported in the present study (**Figure 1**).

**FIGURE 2:**
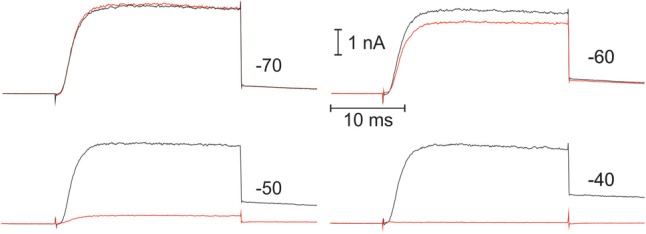
Example of the recording, used for the evaluation of the Kv1.3 equilibrium inactivation as a function of holding voltage. In this experiment with CEM cell, the sample was left for >10 min to equilibrate after breaking into whole cell mode and then current measurements were initiated. At the beginning of each double-pulse sequence, the sample was allowed for 1–2 min to equilibrate at –90 mV and then a first step to +20 mV was applied, evoking large time-dependent current (black trace). After terminating the pulse, the sample was switched for 3 min to a holding voltage, whose value is indicated at the right of each record, and a second pulse to +20 mV was then fired (red trace). The relation between peak current for 2nd and 1st pulses was taken as a measure of the equilibrium inactivation at given holding voltage. Note a very steep voltage dependence for the inactivation.

**FIGURE 3:**
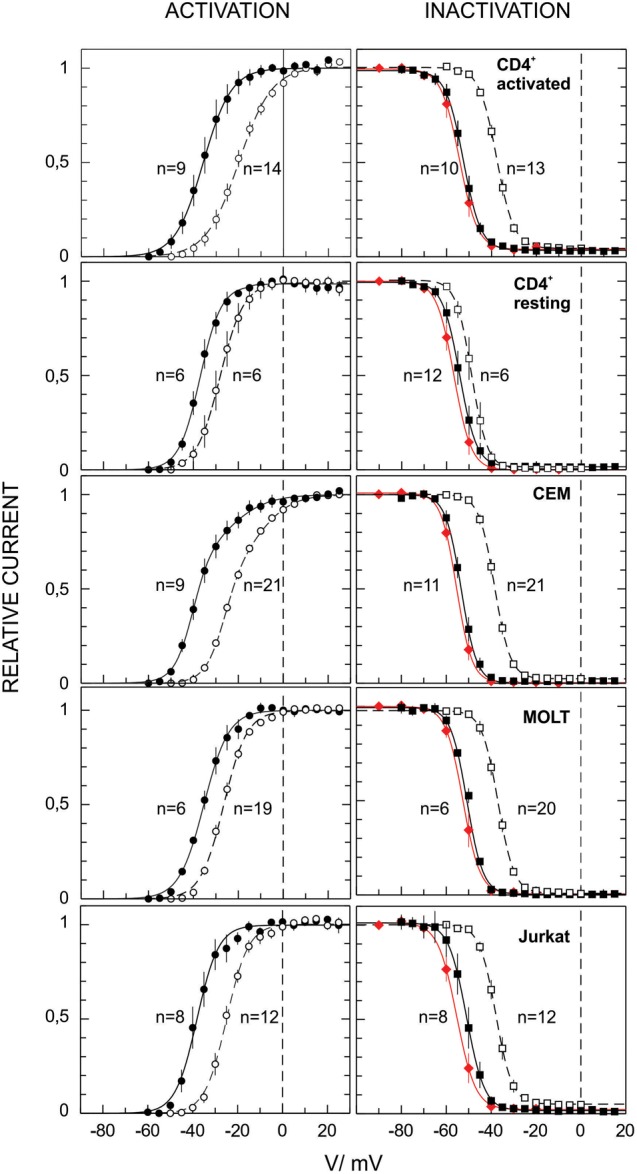
Voltage dependence of the Kv1.3 activation and inactivation in human T lymphocytes and diverse leukemic T cell lines. Activation and inactivation curves were obtained from the experiments as ones presented in **Figure 1** (dashed lines for initial recordings and solid black lines for stable late whole cell recordings) and 2 (red lines, equilibrium inactivation). Inactivation as a function of applied voltage in all cases can be fitted by a single Boltzmann function with parameter values given in **Table 1**. Activation in case of CD4^+^, Jurkat and MOLT-3 cells can be fitted by a single Boltzmann function, with similar parameter values. Voltage-dependence of the Kv activation in CEM cells display an additional component (ca. 1/3 of the total current), with a lesser voltage sensitivity and higher midpoint potential, see **Table 1** for more details.

The voltage-dependence of Kv1.3 current inactivation was obtained by application of rectangular 5 s depolarizing voltage pulses, starting from -60 mV for initial whole cell recording (**Figure 1**, right) or -80 mV after long (>15 min) incubation in the whole cell mode, in 5 mV increments. There were 45 s pauses between consecutive test pulses, to ensure the channels’ recuperation from the inactivation state. To estimate the fraction of a non-inactivated current, short pulse to +50 mV was applied after each test pulse, and respective amplitude was taken relative to a maximum and plotted against the test voltage value (**Figure 3**, right). This relatively short protocol primarily reflects the inactivation, occurring directly from the *open* channel state. It should be noted, that Kv1.3 current is believed to inactivate also from a *closed* state; this process starts at potentials, where the probability of open state is still very low and may proceed with characteristic times as slow as 100 s (Cahalan et al., 1985). Therefore, to evaluate the Kv1.3 channel inactivation at a steady-state, we incubated whole cell samples at -90 mV for >1 min and then apply a pair of depolarizing pulses to +20 mV, separated over 3 min in time by a test holding voltage, ranging between -90 and -20 mV (in 10 mV steps). The degree of equilibrium inactivation as a function of holding voltage was calculated by dividing the amplitude of the peak current evoked by the second pulse through that induced by the first pulse (see a typical experiment in **Figure 2**). For self-explicable reasons such a long protocol may not be used for evaluation of the inactivation process at the beginning of the whole cell recording. Yet, after reaching a stable state of whole cell recording at incubation times >15 min, the results, obtained by the two protocols, short (5 s) and long (3 min) may be compared. As to be expected, a steady-state inactivation curve was shifted to more negative potentials as compared to the curve, obtained by the usage of a shorter voltage protocol. Yet, this shift was relatively small, ranging from 1 to 4 mV, depending on the cell line (**Figure 3**, right, **Table 1**). A small shift may be explained by the fact that the inactivation may occur not directly from the closed state, but via a transient opening. The direct inactivation from open state is a relatively fast process with a characteristic time in the range of 10^-1^ s (**Figure 1**). Multiplying this time by the open/closed equilibrium constant plus 1, one yields the estimate for a characteristic time for the inactivation from a closed state via open one. Thus, when the probability of the Kv1.3 channel to open will approach ∼10% (at voltages above -50 mV, **Figure 3**), the inactivation from a closed state via equilibration with the open state will occur only 10-times slower than a *direct* inactivation from the open state, i.e., it will be essentially completed within 5 s of the test pulse of our short protocol. After correction for a relatively small error in the determination of the midpoint potential, the short protocol as one in **Figure 1**, besides giving direct information on the kinetics of inactivation via the open state, could serve also as a quick estimate of the equilibrium inactivation at any moment.

**Table 1:** Parameters of activation and inactivation of the Kv current in CD4^+^ T lymphocytes and T-ALL cell lines.

Cell line	Activation *V*_1/2_/mV (Z, gating charge)		Inactivation *V*_1/2_/mV (Z, gating charge)		
	Initial	Stable-state	Initial	Stable-state	Stable-state long 2-pulse protocol
CD4^+^ activated	–19.8 ± 0.1 (3.5 ± 0.1)	–35.7 ± 0.3 (4.2 ± 0.2)	–37.8 ± 0.1 (–6.7 ± 0.1)	–52.5 ± 0.1 (–6.6 ± 0.1)	–54.3 ± 0.4 (–6.4 ± 0.3)
CD4^+^ resting	–28.1 ± 0.2 (4.9 ± 0.2)	–37.0 ± 0.2 (5.5 ± 0.2)	–48.9 ± 0.1 (–7.4 ± 0.2)	–54.2 ± 0.1 (–6.8 ± 0.1)	–56.7 ± 0.1 (–6.7 ± 0.1)
CEM^∗^	–26.0 ± 0.4 (5.9 ± 0.3) -9.4 ± 1.7 (3.2 ± 0.4)	–40.4 ± 0.5 (6.4 ± 0.6) -23.4 ± 3.8 (3.0 ± 0.4)	–38.5 ± 0.1 (–7.4 ± 0.1)	–53.4 ± 0.1 (–7.5 ± 0.2)	–55.4 ± 0.1 (–7.5 ± 0.1)
MOLT	–26.5 ± 0.3 (5.0 ± 0.2)	–35.3 ± 0.2 (4.7 ± 0.2)	–37.1 ± 0.1 (–7.2 ± 0.1)	–50.8 ± 0.1 (–7.2 ± 0.1)	–52.6 ± 0.2 (–6.8 ± 0.3)
Jurkat	–24.9 ± 0.3 (5.3 ± 0.2)	–38.1 ± 0.4 (5.4 ± 0.4)	–37.9 ± 0.1 (–7.1 ± 0.1)	–51.2 ± 0.1 (–6.5 ± 0.1)	–55.4 ± 0.2 (–6.2 ± 0.2)

*^∗^In CEM, Kv1.3 activation could be fitted by two Boltzmann components, where the low-potential one (∼2/3) has similar parameter values to those for activation in other T-ALL, whereas the additional high potential one (∼1/3) also displays a lesser voltage sensitivity. Compared to a single Boltzmann fit, two-component Boltzmann curve describes the data for CEM significantly better, with *F*-test yielding *P* = 1.1 × 10^-*4*^ (early whole cell recording) and 2.4 × 10^-*4*^ (at stable state).*

Despite the fact that the Kv current for all leukemic cell lines and healthy T cells was mediated by Kv1.3 channels, its voltage dependence shows some differences (**Figure 3**). Activation curves in case of healthy T lymphocytes, and leukemic Jurkat and MOLT-3 cells could be well described by a single Boltzmann function (Eq. 1 in section “Materials and Methods”). However, in CEM cells single Boltzmann curve provided a poor description of the data. A much better description can be provided by a sum of two different Boltzmann functions (Eq. 2). Single Boltzmann curve describes experimental points equally good with a probability *P* < 0.0003, as yielded by *F*-test. One of the components in the CEM voltage dependence was characterized by very similar parameters values to those obtained for Jurkat, MOLT-3 and resting T lymphocytes; only a midpoint potential for activated T cells has shown a significantly different value in early whole cell recordings (**Table 1**). Activation curves shifted to more negative potentials upon incubation in whole cell mode. At a stable state reached at later times they display little if any difference for healthy lymphocytes and cell lines, but CEM, which preserved a biphasic behavior at all times. The second fraction (about 1/3 of all active Kv1.3 channels) in CEM was characterized by ∼17 mV more positive midpoint potential value and two-times weaker voltage dependence (**Table 1**).

Parameter values for inactivation curves are collected in the **Table 1** and show striking similarity for all leukemic T cell lines used in this study and healthy T cells at a final stable state. However, for early whole cell recordings with resting T cells the midpoint potential value for inactivation appeared to be more negative by ∼10 mV as compared to activated T cells and cell lines. This, however, may be an artifact, dealt with a much smaller size of resting T cells, membrane capacitance of ∼1.7 pF as compared to ∼5 pF for activated T cells and leukemic lymphoblasts. Thus, with resting T cells the washout process would be more rapid so that even early measurements in the whole cell mode might reflect already an altered state. Consequently, with resting T cells substantially smaller relative shifts between initial and final curves were observed both for activation and inactivation, as compared to those with larger cells (**Figure 3**).

### Pharmacological Analysis of the KCa Current Revealed Predominant Functional Expression of KCa3.1 Channels in Healthy T Cells as Well as Leukemic Cell Lines Except Jurkat

KCa current in human T lymphocytes and leukemic T cell line Jurkat is activated by cytosolic Ca^2+^ in a highly cooperative manner, with a full activation at free Ca^2+^ = 1 μM. However, in healthy T cells, either resting or activated, KCa current is sensitive to ChTx, whereas in Jurkat cells respective current is mainly composed by ChTx insensitive KCa channels with a high sensitivity to apamin (Grissmer et al., 1992; Grissmer et al., 1993). Currently it is known that the channels, encoding KCa currents in healthy T and Jurkat cells, are intermediate conductance IKCa (KCa3.1, KCNN4) and small conductance SKCa (KCa2.2, KCNN2) ones (Cahalan and Chandy, 2009). The only report on non-Jurkat leukemic cell line (CEM) suggests that a substantial part of the KCa current in this model is ChTx-sensitive (Zegarra-Moran et al., 1999). Thus, the composition of the KCa current in T leukemic cells is an open issue.

**FIGURE 4:**
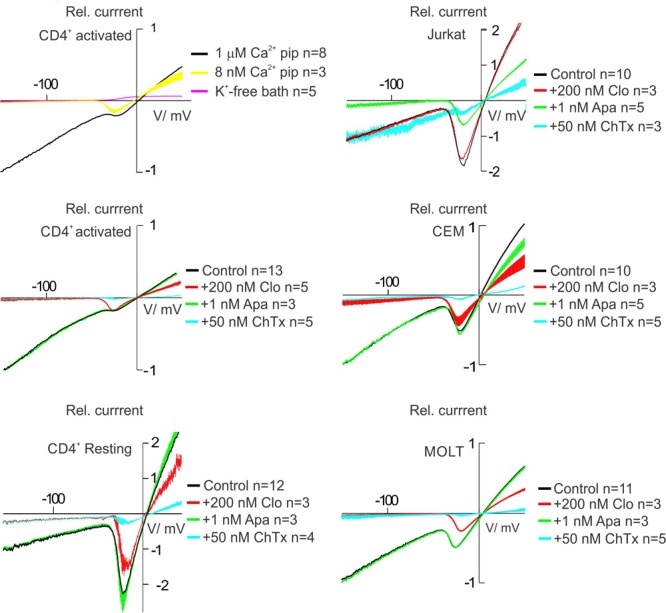
Pharmacology of K^+^ currents in human T lymphocytes and T-ALL cell lines. When no indicated, pipette solution contains 1 μM of free Ca^2+^, allowing recording of both Kv and KCa. To ensure K^+^ selectivity of recorded currents, bath solution was changed to one, where K^+^ was replaced with Na^+^, resulting in undetectable inward current (see example for activated CD4^+^ cells in the upper right corner graph). After recording of a control current, evoked by a voltage-ramp from –150 to + 50 mV (control), one of following drugs was applied to the bath: apamin (1 nM), a specific blocker of KCa 2.2 (SKCa) current, clotrimazole (200 nM) a specific blocker of KCa 3.1 (IKCa) current or charybdotoxin (50 nM), equal potency blocker of KCa 3.1 and Kv1.3 currents. Such experiments with each drug were repeated for at least three separate cells, in every case resulting current was normalized to a control current for the same experiment. See text for more detail and a discussion.

To evaluate KCa current, we have elevated free Ca^2+^ in pipette (intracellular solution) to 1 μM. Yet, Kv1.3 current, unless blocked by some external agent, will be also present under these conditions. To separate Kv1.3 from KCa currents, we applied ramp-wave protocols, starting from very negative potentials (–150 mV), where Kv1.3 channels are closed. To increase the inward current, which at potentials <-40 mV is dominated by KCa, we have increased the external K^+^ concentration. Finally, it was important to keep at minimum the contribution of a non-specific leak current. That it was the case in our experiments is evidenced by following data. First, substitution of K^+^ for Na^+^ in external solution virtually abolished the inward current (**Figure 4**, K-free bath). On contrary, leak conductance does not differentiate between Na^+^ and K^+^, so that inward leak current at -150 mV should be the same in Na^+^ and K^+^ bath. This result also implies that the inward current at -150 mV with high K^+^ bath is mediated by K^+^-selective channels. This K^+^ current is Ca^2+^-activated, because it is abolished at low (8 nM) intracellular Ca^2+^. Second line of evidence for the negligible contribution of leak is the high sensitivity of inward current within -50 to -150 mV range to specific blockers of KCa2.2 or KCa3.1 channels, either to 1 nM apamin (Jurkat) or to 200 nM clotrimazole (other cell lines and healthy T cells), respectively (**Figure 4**). In addition, ChTx, which blocks Kv1.3 and KCa3.1 channels with identical affinity, *K*_d_ ∼ 3.5 nM (Grissmer et al., 1993), at 50 nM concentration produced almost a complete block of the whole cell current, except the case of Jurkat, where voltage-independent component resided. At the same time, the effect of 1 nM apamin on the whole cell current in all cases, but Jurkat, was negligible. The data, presented in **Figure 4** strongly evidence that in cases of human T lymphocytes, CEM and MOLT-3 cell lines KCa current is mediated exclusively by ChTx- and clotrimazole- sensitive KCa3.1 channels. On the contrary, in Jurkat cells we could not detect KCa3.1 channels in the whole cell mode; within a margin of error, the whole cell KCa current in this model was mediated by apamin-sensitive KCa2.2 channels. Although there were some indications in early literature on the presence of low-conductance KCa channels in rat thymus and human T and B cells, there was no evidence on their apamin sensitivity, and, also, in available cases (human T cells) all KCa conductances were sensitive to ChTx (Leonard et al., 1992; Jäger et al., 2000).

### Jurkat Displays Significantly Lower Kv1.3 and KCa Current Densities as Compared to Healthy T Cells and Other T Cell Lines

Specific current densities were evaluated either as early as possible after breaking into whole cell mode (case of Kv1.3), or, in case of KCa few minutes were taken to allowed the perfusion of Ca^2+^ buffer from the pipette to interior of the cell. Joint recording of KCa and Kv currents in whole cell mode reveals large differences in the current density between Jurkat, resting and activated healthy T cells and other T cell lines (**Supplementary Figure S3**). In these records at large negative potentials the whole cell current is dominated by KCa, whereas around -40 mV the Kv activation starts (increase of the inward current); when it reaches its maximum (the minimum of the current), whole cell conductance is defined by the sum of Kv and KCa currents. KCa current density was the highest in activated T cells, followed by MOLT-3 and CEM, it was lower in resting T cells, and the lowest in Jurkat cells. Healthy T cells and all cell lines but Jurkat displayed a relatively large Kv current.

**FIGURE 5:**
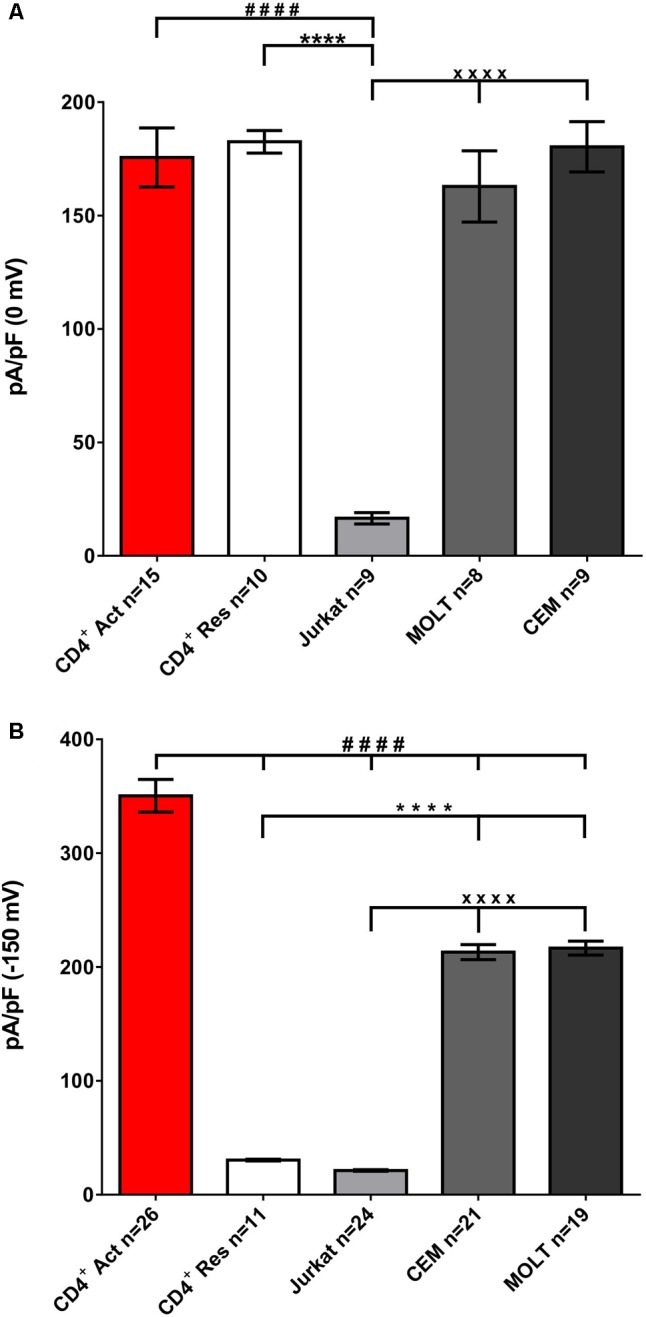
Jurkat displays a 10-fold lower Kv and KCa current density as compared to other cell lines. Specific density of Kv **(A)** and KCa **(B)** current was evaluated at 0 and –150 mV, respectively, in experiments as ones exampled in **Figure 1** and **Supplementary Figure S4**. Data are mean ± SE, with a number of tested cells, *n*, as indicated. Please, mind that Kv = Kv1.3 for all cell lines, whereas KCa is KCa2.2 for Jurkat and KCa3.1 for all other models. Significance was determined by one-tailed test using one-way ANOVA, or unpaired Tuckey *t*-test with Bonferroni correction for multiple comparisons. *P*-values in 5A (Kv): ^####^*p* = 0.00005 (CD4 Act vs. Jurkat); ^∗∗∗∗^*p* = 0.00007 (CD4 Res vs. Jurkat); ^xxxx^*p* = 0.0001 (Jurkat vs. MOLT-3 vs. CEM). *P*-values in 5B (KCa): ^####^*p* = <0.0001 (CD4 Act vs. CD4 Res vs. Jurkat vs. MOLT vs. CEM); ^∗∗∗∗^*p* = 0.00004 (CD4 Res vs. MOLT-3 vs. CEM); ^xxxx^*p* = 0.000038 (Jurkat vs. MOLT-3 vs. CEM).

Quantitative analysis shows that KCa current density increased more than 10-times upon T cells activation (**Figure 5B**). When expressed on the number of channels copies/cell basis, the difference between resting and activated T cells was even larger, 10 and 330 channels/cell, respectively, due to increased cell size upon activation, from 1.7 to 5.1 pF as an average. Density of KCa3.1 current in leukemic T cell lines MOLT-3 and CEM was substantially higher than in resting T cells from healthy individuals and about 62% as an average of that in activated T cells (**Figure 5B**). Irrespective to molecular identity, KCa2.2 in Jurkat vs. KCa3.1 in CEM and MOLT-3, KCa current density in Jurkat was 10 times lower.

When Kv current was assayed separately, at physiological ionic gradients and zero cytosolic Ca^2+^ (as in **Figure 1**), no significant difference in Kv1.3 current density was found between healthy T cells (resting or activated), MOLT-3 and CEM leukemic cell lines; in all cases specific current at 0 mV was between 170 and 180 pA/pF. In Jurkat Kv1.3 current density was 10-times lower (**Figure 5A**). Low functional expression of Kv in Jurkat was also reflected by a more depolarized membrane potential, measured in the current-clamp mode immediately after gaining low resistance access to the cell interior, -20 to -30 mV as compared to -40 to -50 mV in MOLT-3 and CEM cell lines (result not shown).

## Discussion

The significance of K^+^ channels for modulation/maintenance of Ca^2+^ influx in lymphocytes during antigen activation is widely recognized. Two types of K^+^ channels, Kv and KCa, are involved in this function in non-redundant ways: the former via membrane depolarization and the latter in a feedforward manner, via an increase of cytosolic Ca^2+^. Importantly, naïve and T_CM_ cells are characterized by Kv1.3^high^KCa3.1^low^ phenotype, which turns to Kv1.3^high^KCa3.1^high^ upon activation; conversely, T_EM_ cells subset upon activation undergoes a transition from Kv1.3^high^KCa3.1^low^ to Kv1.3^veryhigh^ KCa3.1^low^ phenotype (Wulff et al., 2003). The latter has very important therapeutic consequences for treatment of autoimmune diseases, in particular, multiple sclerosis, mediated by myelin reactive T_EM_, by specific and non-toxic Kv1.3 blockers (reviewed by Panyi, 2005).

In contrast to healthy T lymphocytes, the question about functional expression of K^+^ channels in leukemic cells was not properly addressed yet. For a long time, Jurkat was taken as a prototypic leukemic T cell line. In this study, using patch-clamp technique in whole cell configuration in combination with pharmacological analysis we identified the determinants of Kv and KCa currents in previously unexplored human leukemic cell lines CEM and MOLT-3, and compared their biophysical properties and functional expression pattern with corresponding currents in healthy T cells and well-studied human leukemic cell line Jurkat. All tree cell lines were established from the peripheral blood of T-ALL patients in relapse (Foley et al., 1965; Minowada et al., 1972; Schneider et al., 1977).

Importantly, all experiments in this study were carried out under identical experimental conditions, allowing a detailed quantitative comparison of their Kv and KCa currents. Voltage dependence of Kv1.3 current in all cell lines and resting as well as activated lymphocytes were relatively similar, except that activation of Kv1.3 in CEM cells stably presented additional high potential component with lesser voltage sensitivity (**Table 1**). Most likely, two components with different voltage-activation parameters reflect the presence of two populations of Kv1.3 channels, differed by their local microenvironment. At the same time, these two postulated populations did not differ in the inactivation process. These findings are not contradictory, because Kv1.3 channels undergo P/C-type inactivation, which is due to a conformation change of the selectivity filter (P-gate), deep within a pore, whereas Kv channels opening requires the movement of the A-gate, formed by helices, which cross at cytosolic side, when the channel is closed (reviewed by Bähring et al., 2012). The A-gate, likely, comes to a more intimate contact with the channel protein membrane microenvironment. Values of midpoint potentials for Kv1.3 activation and inactivation for a stable (late) whole cell recordings for all models (**Table 1**) were very close to those, obtained for activated human T cells under identical conditions (Cahalan et al., 1985; Pahapill and Schlichter, 1990). On the other hand, the values obtained at early whole cell measurements or using perforated patch mode (Oleson et al., 1993; Dellis et al., 1999; Hajdú et al., 2003; Pang et al., 2010; Zhao et al., 2015) were more positive and more reminiscent of our initial values, obtained shortly after breaking into whole cell mode here. An up to 15 mV negative shift of the Kv1.3 voltage dependence of activation and inactivation, observed in first 10 min after breaking into whole cell configuration (**Figure 3** and **Table 1**) requires attention. This shift is likely not an artifact as it may not be explained by a dissipation of liquid junction potential between pipette and cell solutions, as confirmed a change of anions from high to low mobile ones in the patch pipette (Cahalan et al., 1985; Chung and Schlichter, 1993). Thus, this shift may be attributed to a gradual washing out of the cytosolic microenvironment of Kv1.3 channels, e.g., affecting the interaction with its Kβ or other auxiliary protein subunits. However, available for the moment data do not give a clear clue on the origin of the observed effect. The oxidation of the Kvβ2 subunit, which is robustly expressed and functionally interacts with the Kv1.3 channel in human T-lymphocytes and T-ALL, causes a rightward shift of the inactivation curve, which could be reversed by the addition of reduced NADPH (Yan et al., 2015). Both activation and inactivation curves of the Kv1.3 in Jurkat cells shifted rightward upon the lipid rafts disruption (Pottosin et al., 2007). Thus, in both cases the effects were opposite to that observed upon the incubation in the whole cell mode. Whatever is the cause of the voltage dependence shift, it has to be taken into the account. It appears that earlier values of voltage dependence parameters may be more useful for the extrapolation to the physiological situation in a living cell. This justifies the usage of shorter voltage protocols, proposed in the present study (**Figure 1**), bearing in mind, however, a small (∼2 mV), but significant difference in the midpoint as compared to a steady-state inactivation (**Table 1**). Notably, activated human T cells display from a very beginning a substantially higher threshold for the channel voltage activation as compared to resting lymphocytes and T-ALL cell lines, but this difference disappears during longer incubation in the whole cell mode (**Figure 3** and **Table 1**). At the same time, the voltage dependence for inactivation was almost identical in activated T cells and leukemic cell lines at any time. Thus, initially, the operation “window,” i.e., the area under the cross between activation and inactivation curves (Pahapill and Schlichter, 1990) is narrower, and the percentage of open and at the same time non-inactivated Kv1.3 channels would be lesser in intact activated T cells (**Figure 3**). This difference may be physiologically important, as it equates to a several-fold decrease in the channel expression.

MOLT-3 and CEM cell lines exhibit Kv1.3^high^KCa3.1^high^ phenotype, fairly comparable to that for activated T cells from healthy donors (**Figure 5**), whereas, in agreement with studies by others, resting T cells exhibited Kv1.3^high^KCa3.1^low^ phenotype (Grissmer et al., 1993; Ghanshani et al., 2000; Wulff et al., 2003). When it comes to Jurkat cells, they represent a very unusual phenotype, with a very low Kv1.3 and also, low KCa, which in this specific case rather than by KCa3.1 was primarily mediated by apamin-sensitive KCa2.2 channels (**Figure 5**). Apamin-sensitive KCa current was present neither in healthy T cells nor in CEM or MOLT-3 (**Figure 4**).

It should be noted that due to their voltage dependence (**Figure 3**), Kv channels, which in T cells and leukemic T cell lines are represented exclusively by Kv1.3, may not clamp membrane voltage to the values below -50 mV. Relative importance of Kv1.3 and KCa in control of membrane potential and Ca^2+^ influx depends also on the context. In resting T cells, where KCa is poorly expressed, membrane potential and Ca^2+^ influx are determined by Kv1.3; conversely, in activated T cells, where KCa3.1 is up-regulated, Ca^2+^ influx is reduced upon the application of KCa3.1 specific blockers and is insensitive to Kv1.3 blockers (Fanger et al., 2001; Panyi, 2005). In Jurkat cells, where both KCa (KCa2.2 in this case) and Kv1.3 are under-expressed (**Figure 5**), Ca^2+^ influx via CRAC depends on KCa and not on Kv1.3. In this case, the role of KCa2.2 is not exclusive, as demonstrated by sequential suppression of KCa2.2 expression and introduction of KCa3.1 (Fanger et al., 2001).

Now, it would be of utterly importance to verify the KvKCa phenotype with leukemic T cells, obtained from patients. Providing these come close to KvKCa profiles in MOLT-3 and CEM, selective KCa3.1 blockers would immediately become a valuable perspective for anti-leukemic therapy. As was evidenced in numerous studies, highly potent KCa3.1 blockers, including clotrimazole and its analogs TRAM-34 and ICA-17043 suppress proliferation of certain types of cancer cells and tumor growth *in vitro*. In contrast to clotrimazole, its derivates TRAM-34 and ICA-17043 do not inhibit cytochrome P450, what makes these compounds more suitable for clinical use (for a review see Chou et al., 2008; for high potency block of KCa3.1 in CEM and MOLT-3 by TRAM-34 see **Supplementary Figure S4**). Because KCa3.1channels are expressed constitutively in salivary glands, intestine, blood vessels and erythrocytes, some adverse effects would be expected, but clinical trials demonstrated that ICA-17043 (Senicapoc^®^) was well tolerated by human voluntaries (Wulff and Castle, 2010).

## Author Contributions

SV-R, IP, and OD: conceived and designed the experiments. GV-C, SV-R, and LL-R: performed the experiments. SV-R and IP analyzed the data and designed the figures. SV-R, GV-C, IP, LL-R, and OD: wrote and/or reviewed the paper. All authors approved the manuscript and are responsible for the accuracy and integrity of any part of the work.

## Conflict of Interest Statement

The authors declare that the research was conducted in the absence of any commercial or financial relationships that could be construed as a potential conflict of interest.

## Supplementary Material

FIGURE S1 Estimates of Kv (Kv 1.3) current density in Jurkat cell line and T cells from the peripheral blood of healthy donors from the published data. When necessary, instead of whole cell current magnitude reported in original study, current density was calculated by dividing whole cell current through published mean capacitance (in pF). In those few studies on Jurkat cells, where mean capacitance was omitted, we used an arbitrary value of 5 pF. Due to a large variation of size, hence, capacitance of activated T cells, only those studies, which report respective cell capacitance were used, the rest of available studies were discarded. Current density was always referred to that at 0 mV as in the present study; in many cases the authors reported mean current density for a distinct voltage (e.g., +40 mV), so we have corrected respective values, using whole cell current-voltage relation for the Kv current extracted from the same study. Median values for Jurkat and healthy T cells are indicated by horizontal lines. Red arrows indicate mean values, obtained in the present study. Following studies were used and indicated by respective symbols: For Jurkat cells: Solé et al., *J. Cell. Sci.* 2016 (○); Conforti et al., *J. Immunol.* 2003 (); Zhao et al., *Cell. Physiol. Biochem.* 2014 (); Lampert et al., *Pflügers Arch.-Eur. J. Physiol.* 2003 (); Zhao et al., *PLoS One* 2013 (); Hosseinzadeh et al., *J. Membr. Biol.* 2015 (); Matsushita et al., *Biochem. Biophys. Res. Comm.* 2008 (); Pottosin et al., *Pflügers Arch.-Eur. J. Physiol.* 2007 (); Szabo et al., *Pflügers Arch.-Eur. J. Physiol.* 1997 (♢); Pang et al., *Biochem. Biophys. Res. Comm.* 2010 (); Chimote et al., *J. Biol. Chem.* 2012 (); Storey et al., *J. Biol. Chem.* 2003 (); Zhao et al., *Sci. Rep.* 2015 (); Bock et al., *Biochem. Biophys. Res. Comm.* 2003 (), Yan et al., *Nanotechnology* 2015 (); Fu et al., *J. Ethnopharmacol.* 2013 (); Kuras et al., *Am. J. Physiol. Cell. Physiol.* (). For healthy T cells: Cahalan et al., *J. Physiol.* 1985 (); Chang et al., *Cell. Physiol. Biochem.* 2001 (); Chung et al., *Pflügers Arch.-Eur. J. Physiol.* 1993 (); Hajdu et al., *Pflügers Arch.-Eur. J. Physiol.* 2003 (); Lee and Deutsch, *Biophys. J.* 1990 (); Oleson et al., *J. Membr. Biol.* 1993 (); Wulff et al., *J. Clin. Invest.* 2003 ().

FIGURE S2 Comparison of activation curves, obtained by tail current analysis or by plotting relative conductance of the Kv 1.3 current. Samples are from the same experiments as in **Figure 3**. To calculate the relative conductance, G, peak of time-dependent current at each voltage was measured and divided through (*V*-*V*_r_), where *V* is clamped voltage and *V*_r_ is the value of reversal potential of the macroscopic K^+^ current (∼-84 mV in this case) and related to the maximal value for every sample, *G*_max_. For a comparison, fits of tail currents with the same parameters as in **Figure 3** (**Table 1**) are presented, for records immediately after breaking into whole cell mode (dashed line) and at a stable state (>15 min in whole cell, solid line).

FIGURE S3 Records of Kv and Ca^2+^-activated K^+^ (KCa) currents in human T lymphocytes and leukemic T cell lines. To facilitate the comparison, current is expressed as a specific current, pA/pF. Pipette contains 1 μM of free Ca^2+^, see text for more details.

FIGURE S4 TRAM-34 potently and with high affinity blocks KCa current in CEM and MOLT-3, but not in Jurkat cells. Examples of whole cell recordings, cell capacity values were 4.5–4.7 pF. Black traces are control records, red traces were recorded after addition of TRAM-34 to a final concentration of 200 nM (Jurkat) of 50 nM (CEM and MOLT-3). Similar results were obtained in three separate experiments with each cell line. Pipette contains 1 μM of free Ca^2+^, for other details of solutions see Section “Materials and Methods.”

## Abbreviations

ChTx: charybdotoxin
CRAC: calcium release activated channel
KCa current: Ca^2+^-activated K^+^ current
Kv current: voltage dependent K^+^ current
MgTx: margatoxin
T-ALL: acute lymphoblastic T leukemia
