# Supplementary figures and images for: Differential Activity of Voltage- and Ca2+-Dependent Potassium Channels in Leukemic T Cell Lines: Jurkat Cells Represent an Exceptional Case
Source: Front Physiol. 2018 May 9;9:499. doi: 10.3389/fphys.2018.00499 (PMC5954129; doi:10.3389/fphys.2018.00499)

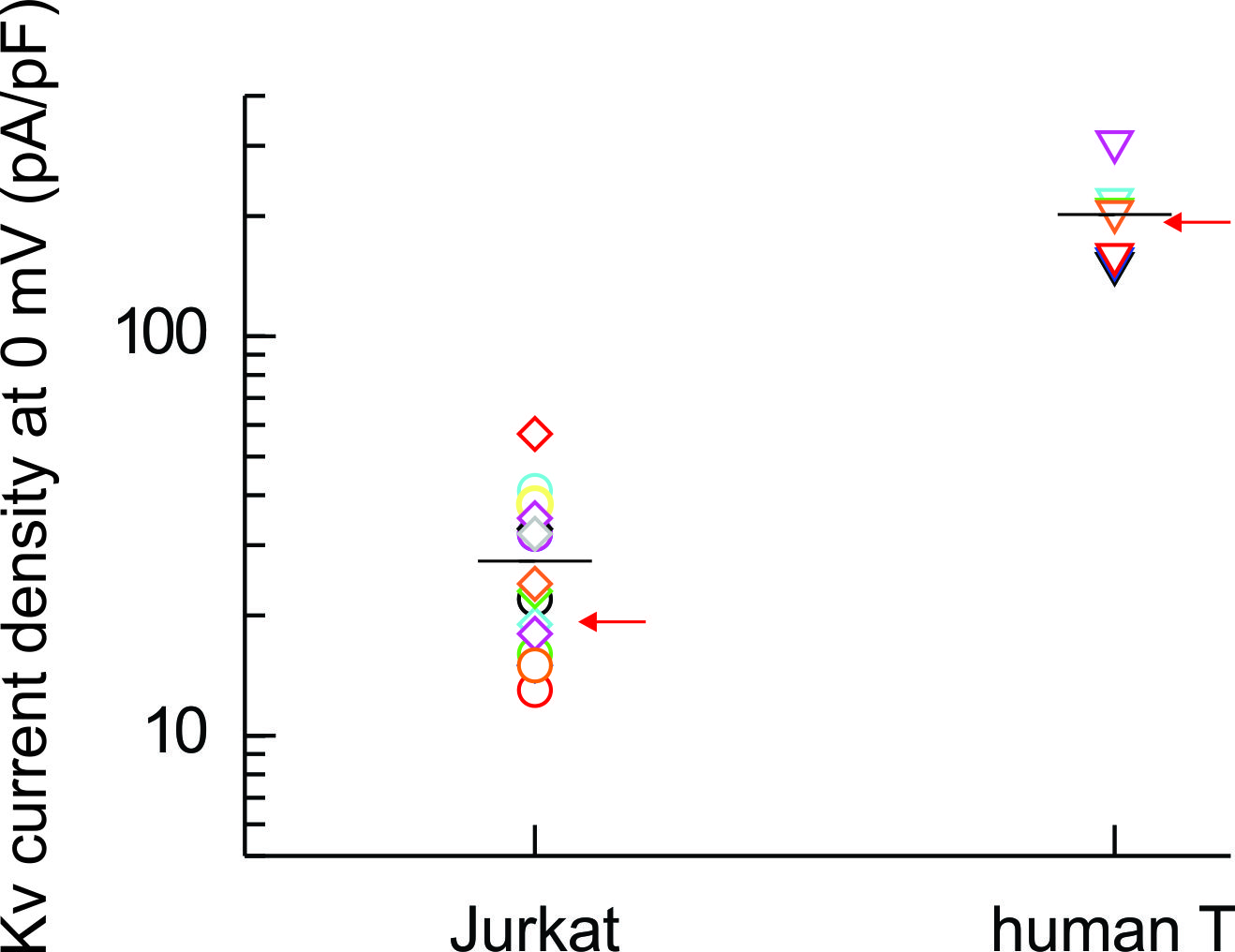

Supplement: FIGURE S1 — Estimates of Kv (Kv 1.3) current density in Jurkat cell line and T cells from the peripheral blood of healthy donors from the published data. When necessary, instead of whole cell current magnitude reported in original study, current density was calculated by dividing whole cell current through published mean capacitance (in pF). In those few studies on Jurkat cells, where mean capacitance was omitted, we used an arbitrary value of 5 pF. Due to a large variation of size, hence, capacitance of activated T cells, only those studies, which report respective cell capacitance were used, the rest of available studies were discarded. Current density was always referred to that at 0 mV as in the present study; in many cases the authors reported mean current density for a distinct voltage (e.g., +40 mV), so we have corrected respective values, using whole cell current-voltage relation for the Kv current extracted from the same study. Median values for Jurkat and healthy T cells are indicated by horizontal lines. Red arrows indicate mean values, obtained in the present study. Following studies were used and indicated by respective symbols: For Jurkat cells: Solé et al., J. Cell. Sci. 2016 (○); Conforti et al., J. Immunol. 2003 (); Zhao et al., Cell. Physiol. Biochem. 2014 (); Lampert et al., Pflügers Arch.-Eur. J. Physiol. 2003 (); Zhao et al., PLoS One 2013 (); Hosseinzadeh et al., J. Membr. Biol. 2015 (); Matsushita et al., Biochem. Biophys. Res. Comm. 2008 (); Pottosin et al., Pflügers Arch.-Eur. J. Physiol. 2007 (); Szabo et al., Pflügers Arch.-Eur. J. Physiol. 1997 (♢); Pang et al., Biochem. Biophys. Res. Comm. 2010 (); Chimote et al., J. Biol. Chem. 2012 (); Storey et al., J. Biol. Chem. 2003 (); Zhao et al., Sci. Rep. 2015 (); Bock et al., Biochem. Biophys. Res. Comm. 2003 (), Yan et al., Nanotechnology 2015 (); Fu et al., J. Ethnopharmacol. 2013 (); Kuras et al., Am. J. Physiol. Cell. Physiol. (). For healthy T cells: Cahalan et al., J. Physiol. 1985 (); Chang et al., [file Image_1.JPEG]

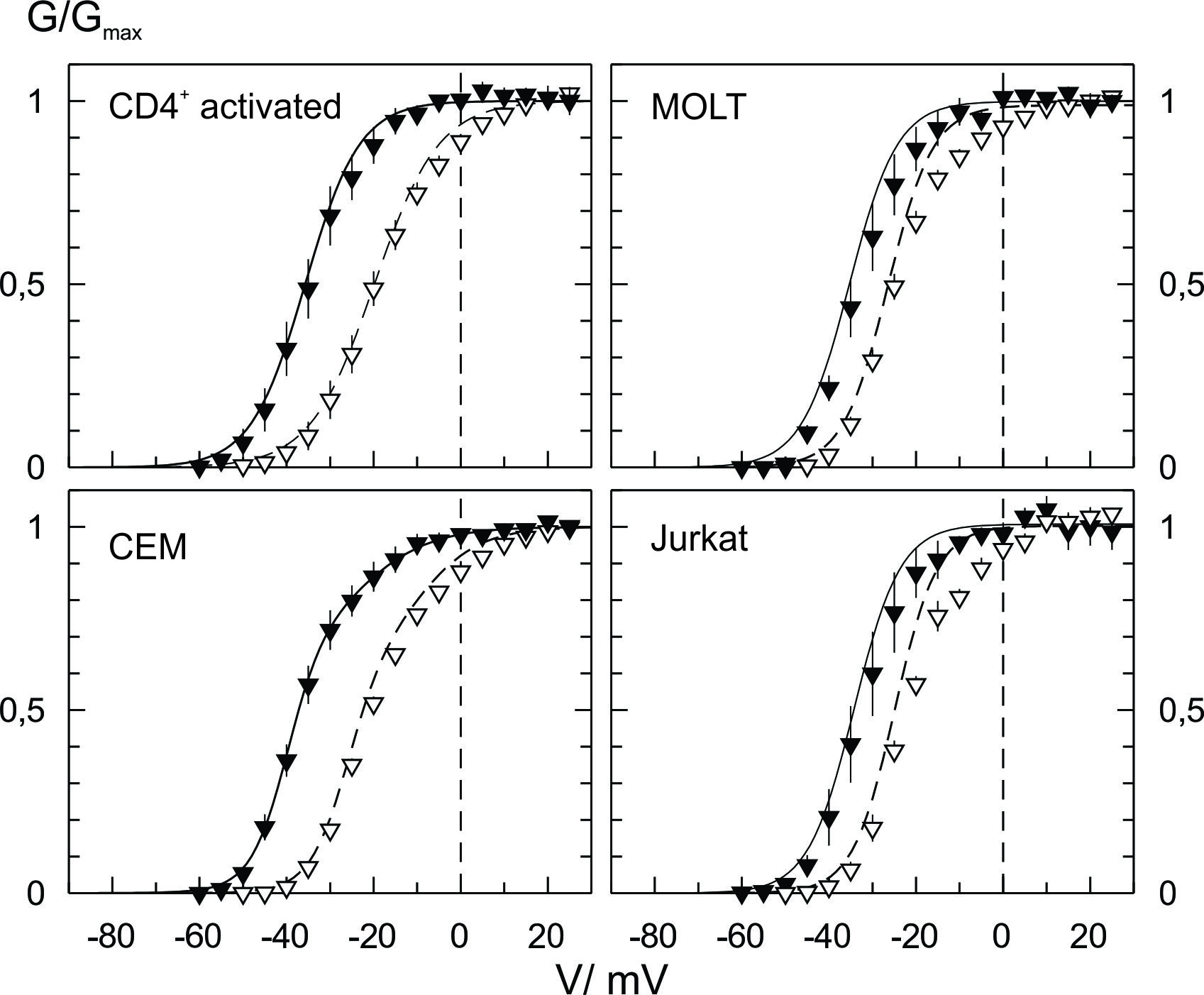

Supplement: FIGURE S2 — Comparison of activation curves, obtained by tail current analysis or by plotting relative conductance of the Kv 1.3 current. Samples are from the same experiments as in Figure 3. To calculate the relative conductance, G, peak of time-dependent current at each voltage was measured and divided through (V-Vr), where V is clamped voltage and Vr is the value of reversal potential of the macroscopic K+ current (∼-84 mV in this case) and related to the maximal value for every sample, Gmax. For a comparison, fits of tail currents with the same parameters as in Figure 3 (Table 1) are presented, for records immediately after breaking into whole cell mode (dashed line) and at a stable state (>15 min in whole cell, solid line). [file Image_2.JPEG]

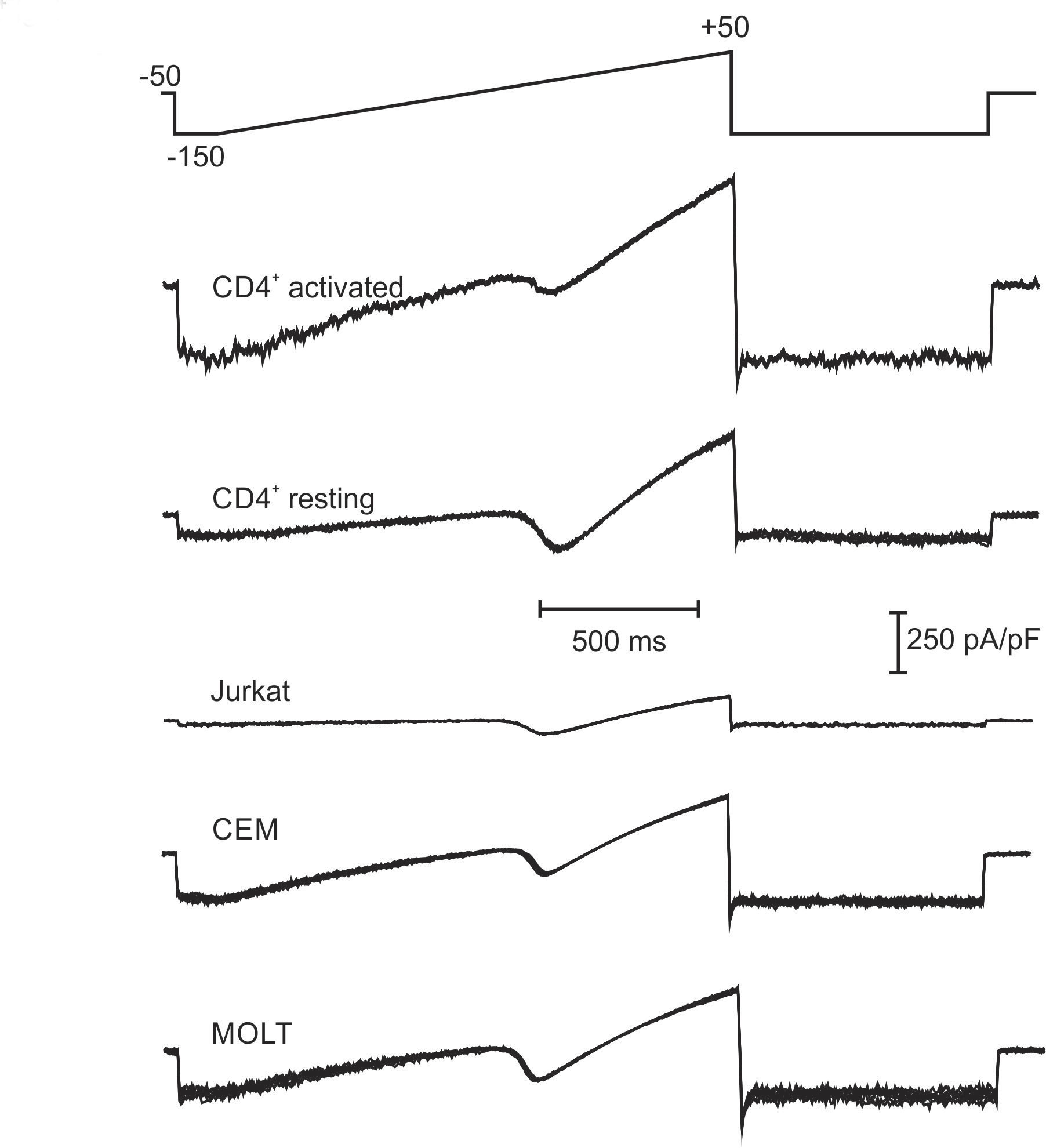

Supplement: FIGURE S3 — Records of Kv and Ca2+-activated K+ (KCa) currents in human T lymphocytes and leukemic T cell lines. To facilitate the comparison, current is expressed as a specific current, pA/pF. Pipette contains 1 μM of free Ca2+, see text for more details. [file Image_3.JPEG]

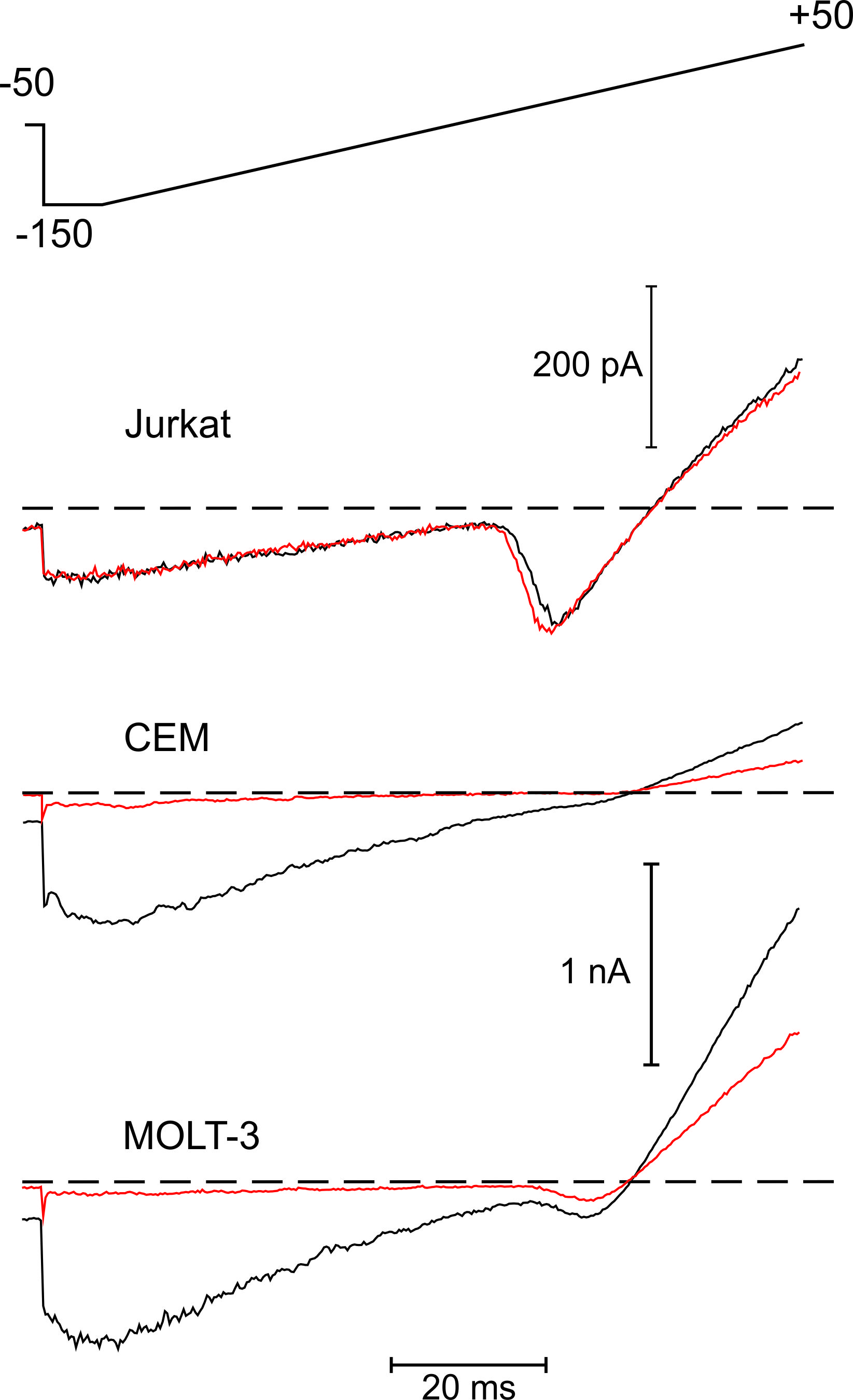

Supplement: FIGURE S4 — TRAM-34 potently and with high affinity blocks KCa current in CEM and MOLT-3, but not in Jurkat cells. Examples of whole cell recordings, cell capacity values were 4.5–4.7 pF. Black traces are control records, red traces were recorded after addition of TRAM-34 to a final concentration of 200 nM (Jurkat) of 50 nM (CEM and MOLT-3). Similar results were obtained in three separate experiments with each cell line. Pipette contains 1 μM of free Ca2+, for other details of solutions see Section “Materials and Methods.” [file Image_4.JPEG]
